# Bilateral Optic Neuritis as the Initial Presentation of Acute HIV Infection in a Young Bodybuilder: A Case Report

**DOI:** 10.3390/reports9010052

**Published:** 2026-02-03

**Authors:** Jennifer Lu, Kathleen Tor, Joseph Yasmeh, Monique George

**Affiliations:** Department of Family Medicine, Kaiser Permanente Woodland Hills, Woodland Hills, CA 91367, USA

**Keywords:** optic neuritis, HIV, case report

## Abstract

**Background and Clinical Significance**: Optic neuritis, an inflammatory demyelinating condition affecting the optic nerve, can present as an isolated phenomenon or as a harbinger of an underlying systemic disorder. While often associated with multiple sclerosis (MS), optic neuritis has been reported in the context of various infectious and inflammatory conditions, including human immunodeficiency virus (HIV) infection. **Case Presentation**: We describe a case of bilateral optic neuritis that led to the diagnosis of acute HIV infection in a young man actively engaged in bodybuilding, anabolic steroid use, and high-risk sexual behavior. The patient initially presented with an acute onset of vision loss, headache, and photophobia. Magnetic resonance imaging (MRI) findings confirmed demyelination of the optic nerves, and laboratory workup revealed acute HIV-1 infection. The patient improved following high-dose corticosteroids and initiation of antiretroviral therapy. **Conclusions**: This case highlights the need to consider systemic infections such as HIV in atypical presentations of optic neuritis.

## 1. Introduction and Clinical Significance

Optic neuritis is an inflammatory demyelinating disorder of the optic nerve that is classically associated with multiple sclerosis (MS); however, there are various systemic, infectious, and autoimmune causes of optic neuritis [1,2]. Bilateral optic neuritis, especially in the absence of established MS or opportunistic infection, is an uncommon presentation that should prompt clinicians to consider systemic infectious etiologies, including acute human immunodeficiency virus (HIV) infection [3,4,5]. HIV is a less frequently recognized cause of optic neuritis, and the pathophysiology of HIV-associated optic neuropathy remains incompletely understood. Differing hypotheses propose direct viral invasion, secondary inflammatory demyelination, or vascular dysregulation as potential mechanisms. Early recognition and diagnosis are crucial as timely initiation of corticosteroid therapy and antiretroviral treatment may improve visual and neurological outcomes [3,4]. 

This case report describes bilateral optic neuritis as the initial manifestation of acute HIV infection in a young man with significant anabolic steroid exposure and high-risk sexual behavior. This report highlights diagnostic challenges, underscores the importance of maintaining a broad differential diagnosis in atypical optic neuritis presentations, and emphasizes the interplay between HIV, immune dysregulation, and coexisting vascular risk factors. It demonstrates the need for clinicians to maintain a broad differential when evaluating optic neuritis, particularly when features are bilateral, atypical, or unexplained by more common etiologies.

## 2. Case Presentation

A 38-year-old man presented to the emergency department with a one-week history of progressive vision loss predominantly affecting the right eye, accompanied by a severe frontal headache and photophobia. His right-sided blurred vision had been worsening over the week prior to presentation, and by the day before presentation, he reported complete vision loss of the right eye. Additionally, on the day of presentation, he also reported decreased color vision of the left eye. 

The patient’s medical history was notable for hypertension, severe obstructive sleep apnea, and episodic migraine-like headaches with visual aura for the past year. He reported inconsistent use of continuous positive airway pressure therapy for his sleep apnea. His social history was notable for high-risk sexual behavior, including unprotected sexual encounters with both male and female partners. He reported a two-year history of self-administering intramuscular anabolic steroids (testosterone and nandrolone decanoate) with sterile needles for body-building purposes. He denied intravenous drug use.

His vital signs upon presentation were notable for an elevated blood pressure of 149/92 mmHg; the remainder of his vitals were within normal limits: he had a heart rate of 89 beats per minute, temperature of 36.7 °C (98.1 °F), respiratory rate of 19 breaths per minute, and oxygen saturation level of 98% at room air. On examination, the patient’s visual acuity was markedly impaired, with a visual acuity of 20/200 in the right eye and 20/40 in the left eye. The right pupil was dilated and minimally reactive to light, while the left pupil responded normally. Extraocular movements were intact bilaterally, and fundoscopic examination revealed normal appearing optic discs without evidence of papilledema or other abnormalities. The remainder of his neurological examination was unremarkable.

**Figure 1:**
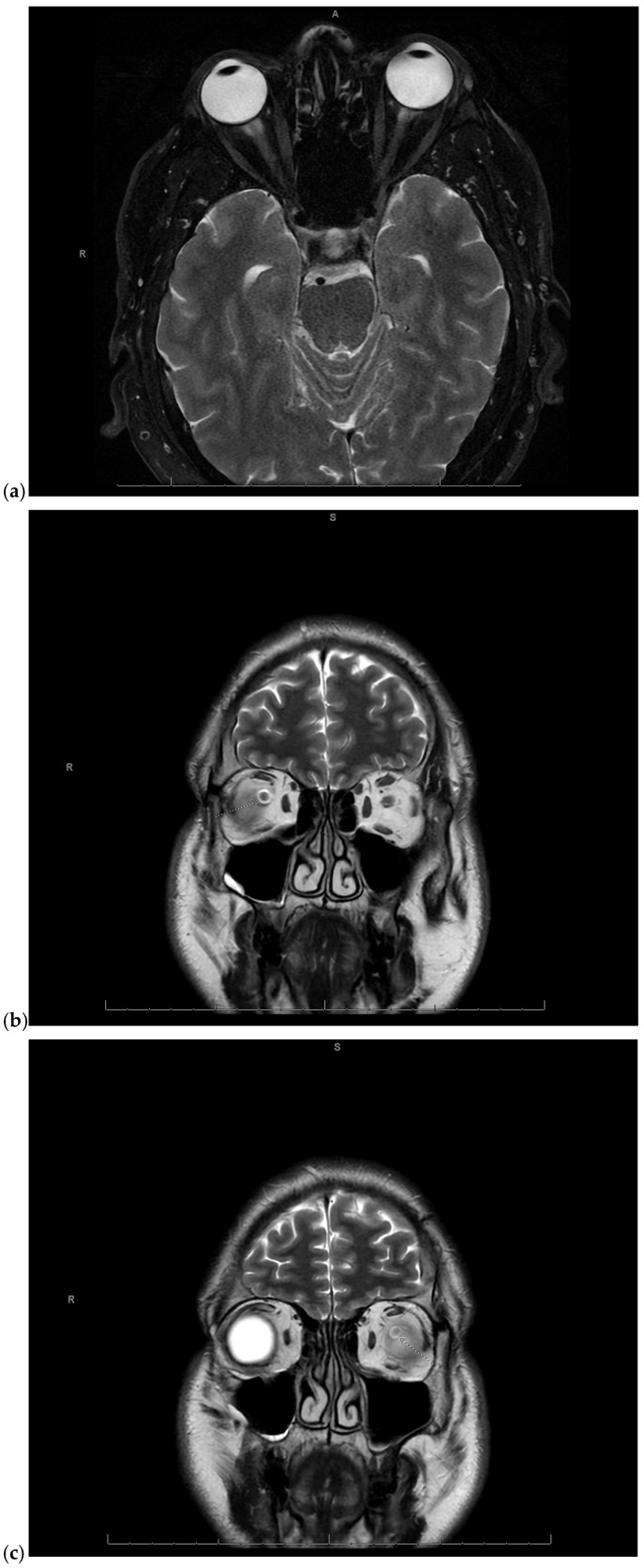
(**a**) Magnetic Resonance Imaging (MRI) of the brain and orbits fat-saturated T2-weighted axial image showing mild right greater than left T2 hyperintense signal in the periphery of the optic nerves in the anterior intraorbital region, consistent with optic neuritis. A: anterior, R: right; (**b**) MRI of the brain and orbits coronal T2-weighted image showing right sided optic neuritis. S: superior, R: right; (**c**) MRI of the brain and orbits coronal T2-weighted image showing left sided optic neuritis. S: superior, R: right.

Given the concerning visual symptoms, the patient underwent an emergent magnetic resonance imaging (MRI) of the brain and orbits, which revealed T2 hyperintense signal abnormalities in the periphery of the optic nerves bilaterally, more pronounced on the right side, consistent with optic neuritis (Figure 1a–c).

To investigate potential etiologies, further workup was pursued. Laboratory studies were remarkable for leukocytosis (WBC 19.7 × 1000/mcL), hypertriglyceridemia (190 mg/dL), and a low high-density lipoprotein (HDL) level (34 mg/dL). The myelin oligodendrocyte glycoprotein (MOG) antibody IgG and aquaporin-4 (AQP4) receptor IgG were both negative. A lumbar puncture was performed, and cerebrospinal fluid (CSF) analysis revealed a lymphocytic pleocytosis (3–4 cells/mm^3^ with 82–90% lymphocytes) and elevated glucose level (82 mg/dL), but was otherwise unremarkable, with negative bacterial, viral, and fungal studies and a negative multiple sclerosis panel (Table 1). Additional imaging studies, including MRI of the cervical, thoracic, and lumbar spine, as well as a computed tomography (CT) scan of the chest, did not reveal any evidence of demyelinating lesions or granulomatous disease. CT angiography of the head and neck demonstrated severe intracranial stenoses, an unusual finding for the patient’s age. The patient’s HIV-1/2 antigen/antibody combination assay returned reactive, and confirmatory HIV-1 antibody testing was positive, indicating a new diagnosis of HIV-1 infection (Table 1).

**Figure 2:**
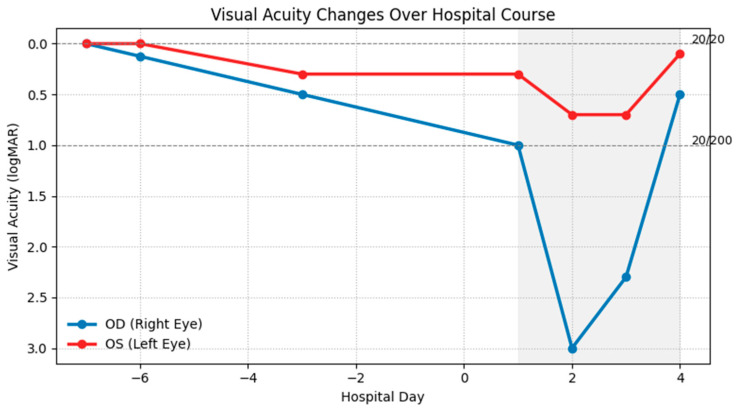
Visual acuity in the right (OD, blue) and left (OS, red) eyes is shown in Logarithm of the Minimum Angle of Resolution (logMAR) units on the *y*-axis (lower values indicate better vision). Hospital days are shown on the *x*-axis; the shaded region indicates hospitalization, during which high-dose intravenous steroids were administered daily. The right eye declined rapidly from baseline 20/20 to 20/200 on admission, reaching no light perception before partial recovery, while the left eye showed a milder decline with earlier stabilization and gradual improvement.

Given the bilateral optic neuritis, the patient was promptly initiated on high-dose intravenous methylprednisolone (1 g daily) for five days, as recommended by neurology and ophthalmology consultations. His visual symptoms began to improve by the third day of treatment, and he had partial recovery of vision in the right eye and restoration of color perception in the left eye on the fourth day of treatment (Figure 2). Discussions were held regarding the potential need for additional immunomodulatory therapies for optic neuritis, such as plasmapheresis or rituximab. However, these interventions were deferred in light of his clinical improvement.

**Figure 3:**
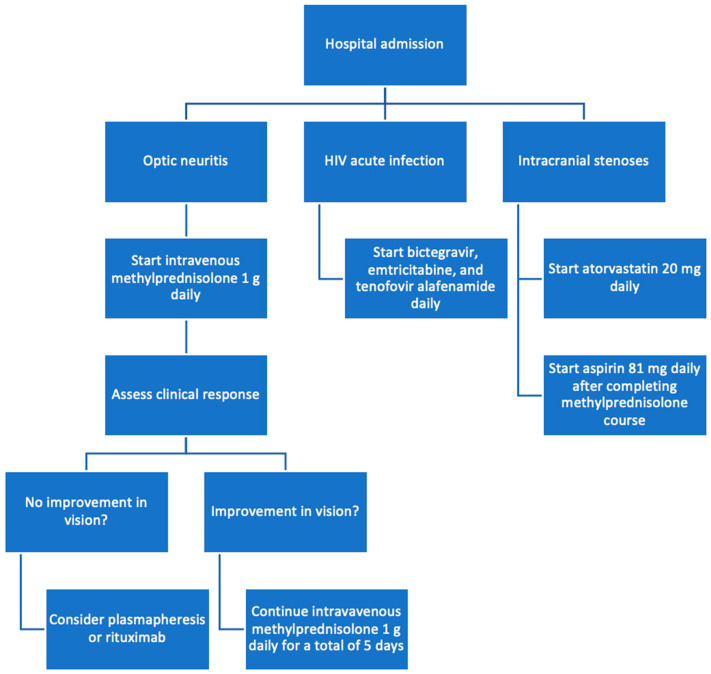
Flowchart of treatment process.

After consultation with infectious disease, antiretroviral therapy with a fixed-dose combination of bictegravir, emtricitabine, and tenofovir alafenamide (Biktarvy) was initiated for the management of the patient’s newly diagnosed HIV-1 infection. Given the unusual finding of severe intracranial stenoses in a young patient, neurology was consulted and attributed this vascular pathology to the patient’s prolonged anabolic steroid use. He was counseled to discontinue the use of anabolic steroids immediately and was started on aspirin and atorvastatin for vascular protection and risk reduction (Figure 3).

The patient was discharged home with close outpatient follow-up with ophthalmology, infectious disease, and neurology to monitor his visual recovery, the management of his HIV infection, and his neurological status.

## 3. Discussion

This case highlights the importance of maintaining a broad differential diagnosis when evaluating patients with optic neuritis, as it can be the presenting manifestation of various underlying systemic conditions. The most common causes of bilateral optic neuritis in younger adults include MS, myelin oligodendrocyte glycoprotein antibody-associated disease (MOGAD), and neuromyelitis optica spectrum disorder, aquaporin-4 IgG positive (NMOSD, AQP4-IgG positive) [6,7]. However, optic neuritis can also occur in the context of infectious and inflammatory disorders, including HIV infection [1]. In the setting of HIV, the pathophysiology of optic neuritis is not completely understood and requires further research. Potential mechanisms include direct viral infection of the optic nerve, opportunistic infections, immune reconstitution inflammatory syndrome (IRIS) following the initiation of antiretroviral therapy, or as part of an HIV-associated neuroretinal disorder [3]. 

The patient’s risk factors for HIV acquisition, including unprotected sexual encounters and anabolic steroid use (which has been associated with increased risk-taking behavior), likely contributed to HIV acquisition and prompted HIV testing as part of the diagnostic workup. Because HIV staging can materially affect etiologic interpretation, we note that the timing of acquisition cannot be conclusively determined from the available laboratory snapshot alone. HIV ribonucleic acid (RNA) levels during acute infection are often very high but can decline over subsequent weeks toward a lower set point, and this patient’s antibody positivity indicates that seroconversion has already occurred [8]. Thus, other immune-mediated demyelinating disorders were key considerations in this patient’s bilateral optic neuritis, particularly MOGAD and NMOSD, AQP4-IgG positive, both of which may present with marked visual loss and respond to high-dose corticosteroids [4]. However, given the negative MOG antibody IgG and AQP4 receptor IgG, as well as the similarities of this case to several reports which describe bilateral optic neuritis presenting in the setting of HIV infection, including during acute HIV/seroconversion, HIV remained the most likely contributor to this patient’s optic neuritis. Fortin et al. reported bilateral optic neuritis attributed to acute HIV infection, illustrating that severe bilateral inflammatory optic neuropathy can occur early in HIV and may overlap clinically with other demyelinating phenotypes [5]. Larsen et al. similarly described bilateral optic neuritis in acute HIV infection, reinforcing that optic neuritis can coincide with early HIV disease states in the published literature [9]. Our case shares an atypical bilateral presentation with MRI findings consistent with optic neuritis and inflammatory CSF features, with clinical improvement after high-dose corticosteroids and initiation of antiretroviral therapy [5,9].

Furthermore, the patient’s prolonged use of anabolic steroids was implicated in the development of severe intracranial stenoses, an unusual finding for his age. Anabolic-androgenic steroids (AAS) have been linked to endothelial dysfunction, accelerated atherosclerosis, and an increased risk of cerebrovascular events, providing a plausible vascular pathway for intracranial arterial disease [10]. AAS use negatively impacts lipid profiles, most evidently with doses that exceed physiologic levels [11]. AAS increases low-density lipoprotein (LDL) cholesterol by over 50% and decreases HDL cholesterol by over 30%. Although direct evidence linking AAS use to intracranial stenosis remains limited, it has been demonstrated that AAS promotes systemic atherosclerosis in a dose-dependent manner [12]. This suggests similar processes may affect intracranial arteries over time. Thus, the prompt initiation of antiplatelet and statin therapy was warranted to mitigate further vascular complications. However, intracranial arterial stenosis may be multifactorial; cerebral arterial disease has also been described in HIV cohorts across a range of presentations, and HIV-related vascular injury (direct or indirect) cannot be excluded purely on the basis of age or a single immune snapshot [13]. Other non-atherosclerotic causes of intracranial stenosis in young adults include inflammatory vasculopathies, Moyamoya disease, arterial dissection, reversible cerebral vasoconstriction syndrome, fibromuscular dysplasia, or antiphospholipid antibody syndrome with which the work-up in this case was inconsistent [14,15].

AAS exposure may also be relevant from an immunologic standpoint. Androgens can exert immunomodulatory effects across innate and adaptive compartments, and high-dose anabolic steroids have been reported to alter immune responses in experimental models [16,17]. These mechanisms raise the possibility that supraphysiologic AAS exposure could influence immune signaling during HIV infection and potentially affect the clinical expression of inflammatory complications such as optic neuritis. However, clinical data directly linking non-prescribed AAS use to greater severity of acute/recent HIV infection, or to worse outcomes in HIV-associated optic neuritis, are sparse; accordingly, this is presented as biologic plausibility rather than a causal claim.

Published management of HIV-associated optic neuritis is heterogeneous and largely limited to case reports and small series. Reported strategies commonly include high-dose intravenous corticosteroids for acute optic neuritis, initiation of antiretroviral therapy when HIV is newly diagnosed or uncontrolled, and targeted antimicrobial therapy when coinfections are implicated [2,3,5,9]. Visual outcomes appear variable, with multiple reports describing partial to substantial improvement, while delayed diagnosis or alternative mechanisms may be associated with poorer recovery [5,9]. In refractory or severe optic neuritis phenotypes, escalation therapies such as plasmapheresis may be considered in optic neuritis paradigms, although HIV-associated optic neuritis-specific outcome data remain limited [2]. Given the overlap between HIV-associated optic neuritis and other immune-mediated demyelinating etiologies, standardized reporting of MOGAD and NMOSD, AQP4-IgG positive relevant testing, imaging characteristics, and treatment would strengthen future comparisons [18,19].

The management of this patient’s condition involved a multidisciplinary approach, incorporating high-dose corticosteroid therapy for the optic neuritis, initiation of antiretroviral therapy for newly diagnosed HIV infection, and appropriate vascular risk reduction measures for the intracranial stenoses. Close outpatient follow-up with ophthalmology, infectious disease, and neurology is essential to monitor visual recovery, optimize HIV and vascular risk management, and reassess etiology if relapse or new neurologic symptoms occur [2].

## 4. Conclusions

This case underscores the importance of maintaining a broad differential diagnosis when evaluating patients with optic neuritis and highlights the potential for HIV infection to manifest with neurological complications, even in the absence of overt systemic symptoms. The prompt recognition of this patient’s condition, coupled with a coordinated multidisciplinary approach to management, was crucial in addressing the various aspects of his presentation and optimizing his overall care.

## Figures and Tables

**Table 1:** Pertinent serum laboratory results and all cerebrospinal fluid (CSF) laboratory results. Abnormal results bolded.

Lab Test	Source	Result	Reference Range
White Blood Cells	Blood	**19.7 × 1000/mcL**	4.0–11.0 × 1000/mcL
Hemoglobin	Blood	16.4 g/dL	13.5–17.5 g/dL
Platelets	Blood	341 × 1000/mcL	130–400 × 1000/mcL
Total Cholesterol	Blood	168 mg/dL	≤199 mg/dL
Triglycerides	Blood	**190 mg/dL**	≤149 mg/dL
High Density Lipoprotein	Blood	**34 mg/dL**	≥40 mg/dL
Low Density Lipoprotein	Blood	**101 mg/dL**	≤99 mg/dL
HIV 1/2 Ab + p24 Ag Screen	Blood	**Reactive**	Nonreactive
HIV 1/2 Differentiation Assay	Blood	**HIV-1 Positive**	HIV Negative
HIV-1 RNA PCR	Blood	**1490 copies/mL**	≤0
HIV-1 RNA Log	Blood	**3.17 log copies/mL**	≤0.00
CD3+ T Cells %	Blood	61.06%	56.86–82.50%
CD3+ T Cells	Blood	**611 cells/uL**	823–2547 cells/uL
CD4+CD3+ T Cells %	Blood	**31.74%**	32.42–63.19%
CD4+CD3+ T Cells	Blood	**317 cells/uL**	488–1711 cells/uL
CD8+CD3+ T Cells %	Blood	29.67%	8.99–38.99%
CD8+CD3+ T Cells	Blood	297 cells/uL	154–1097 cells/uL
CD4/CD8 Ratio	Blood	1.07	≥0.90
Myelin Oligodendrocyte Glycoprotein (MOG) Antibody IgG	Blood	<1:10	<1:10
Aquaporin-4 (AQP4) Receptor IgG	Blood	Negative	Negative
CSF Appearance	CSF	Clear	Clear
CSF Color	CSF	Colorless	Colorless
Xanthochromia	CSF	Negative	Negative
RBC Count	CSF	**158 cells/mm^3^**	≤0 cells/mm^3^
White Blood Cell Count	CSF	4 cells/mm^3^	0–5 cells/mm^3^
Nucleated Cells	CSF	3–4 cells/mm^3^	0–5 cells/mm^3^
Neutrophils %	CSF	11%	Not specified
Lymphocytes %	CSF	**82–90%**	40–80%
Monocytes/Macrophages %	CSF	7%	Not specified
Eosinophils %	CSF	0%	≤0%
Basophils %	CSF	0%	≤0%
Oligoclonal Bands	CSF	Absent	Absent
Glucose	CSF	**82 mg/dL**	50–75 mg/dL
Protein	CSF	32 mg/dL	15–45 mg/dL
Albumin	CSF	16.3 mg/dL	8.0–42.0 mg/dL
Albumin	Blood	4.7 g/dL	3.6–5.1 g/dL
IgG	CSF	2.9 mg/dL	0.8–7.7 mg/dL
IgG	Blood	1540 mg/dL	600–1640 mg/dL
IgG Index	CSF	0.54	<0.70
IgG Synthesis Rate	CSF	−3.5 mg/24 h	−9.9 to +3.3 mg/24 h
Venereal Disease Research Laboratory	CSF	Non-Reactive	Non-Reactive
Cryptococcal Antigen	CSF	Negative	Negative
Angiotensin Converting Enzyme Level	CSF	<5 U/L	≤15 U/L
CSF Culture	CSF	No growth at 3 days	No growth
*E. coli* K1 DNA	CSF	Not Detected	Not Detected
*H. influenzae* DNA	CSF	Not Detected	Not Detected
*Listeria monocytogenes* DNA	CSF	Not Detected	Not Detected
*Neisseria meningitidis* DNA	CSF	Not Detected	Not Detected
*Strep agalactiae* DNA	CSF	Not Detected	Not Detected
*Strep pneumoniae* DNA	CSF	Not Detected	Not Detected
CMV DNA	CSF	Not Detected	Not Detected
Enterovirus RNA	CSF	Not Detected	Not Detected
HSV-1 DNA	CSF	Not Detected	Not Detected
HSV-2 DNA	CSF	Not Detected	Not Detected
HHV-6 DNA	CSF	Not Detected	Not Detected
VZV DNA	CSF	Not Detected	Not Detected
Parechovirus RNA	CSF	Not Detected	Not Detected
*Cryptococcus gattii/neoformans* DNA	CSF	Not Detected	Not Detected
*Borrelia burgdorferi* IgG	CSF	No bands detected	Negative
*Borrelia burgdorferi* IgM	CSF	No bands detected	Negative
*Coccidioides* IgG	CSF	Negative	Negative
*Coccidioides* IgM	CSF	Negative	Negative

## Data Availability

The data presented in the study are available on request from the corresponding author due to privacy concerns.

## Abbreviations

The following abbreviations are used in this manuscript:

MS	Multiple sclerosis
HIV	Human immunodeficiency virus
MRI	Magnetic resonance imaging
HDL	High-density lipoprotein
MOG	Myelin oligodendrocyte glycoprotein
AQP4	Aquaporin-4
CSF	Cerebrospinal fluid
CT	Computed tomography
logMAR	Logarithm of the Minimum Angle of Resolution
MOGAD	Myelin oligodendrocyte glycoprotein antibody–associated disease
NMOSD	Neuromyelitis optica spectrum disorder
IRIS	Immune reconstitution inflammatory syndrome
RNA	Ribonucleic acid
AAS	Anabolic-androgenic steroid
LDL	Low-density lipoprotein
